# Supplementary material for: Inventory statistics meet big data: complications for estimating numbers of species
Source: PeerJ. 2020 May 13;8:e8872. doi: 10.7717/peerj.8872 (PMC7229767; doi:10.7717/peerj.8872)
Supplement: Supplemental Information 1 [file peerj-08-8872-s001.docx]

**SUPPORTING INFORMATION**

The following three pages offer results of analyses of simulations using three other inventory statistics (jack2, jack1, ICE), as well as Chao2 under two additional parameterizations of log-normal and three additional parameterizations of gamma distributions, showing similar results to those presented in Figure 3 and Figure 4.


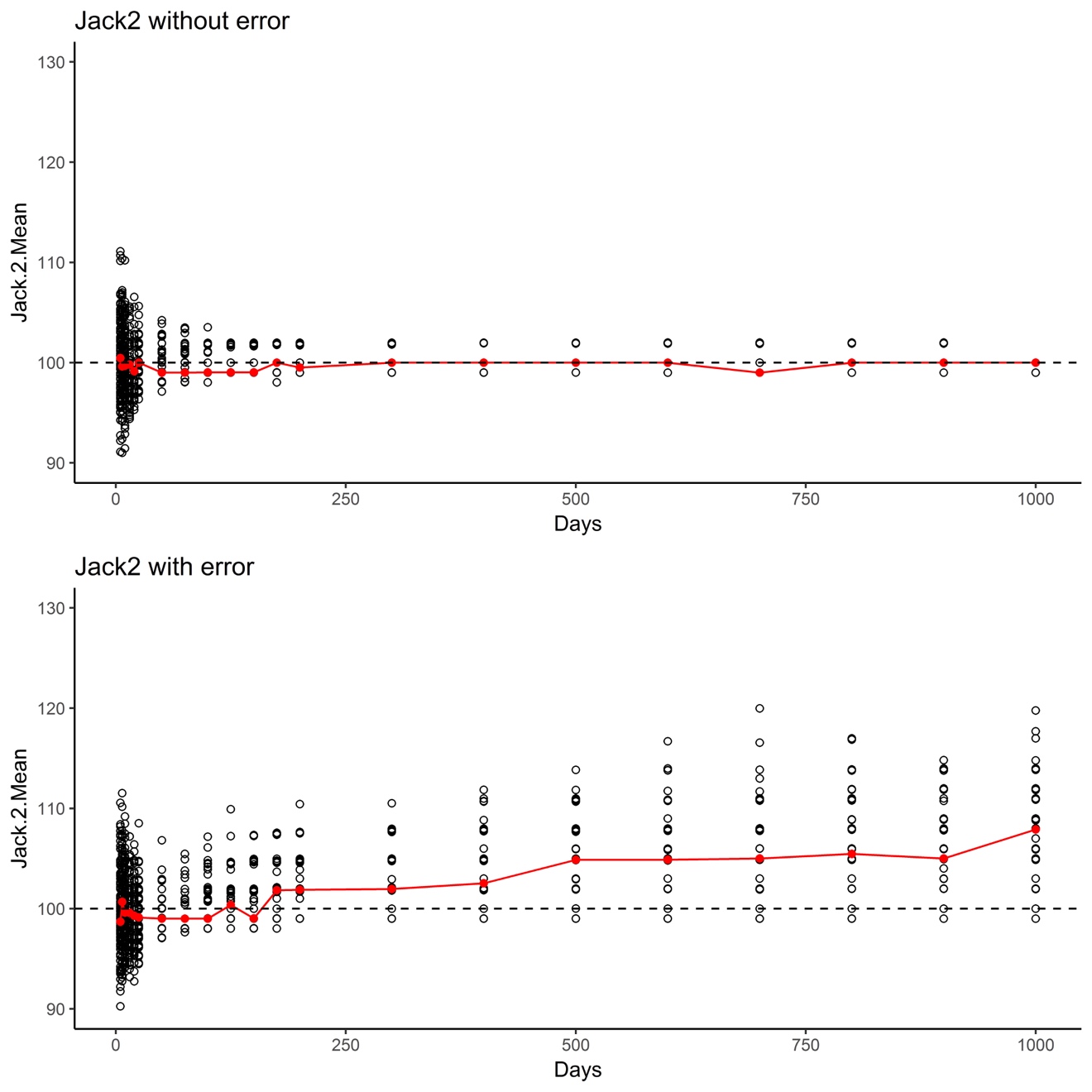

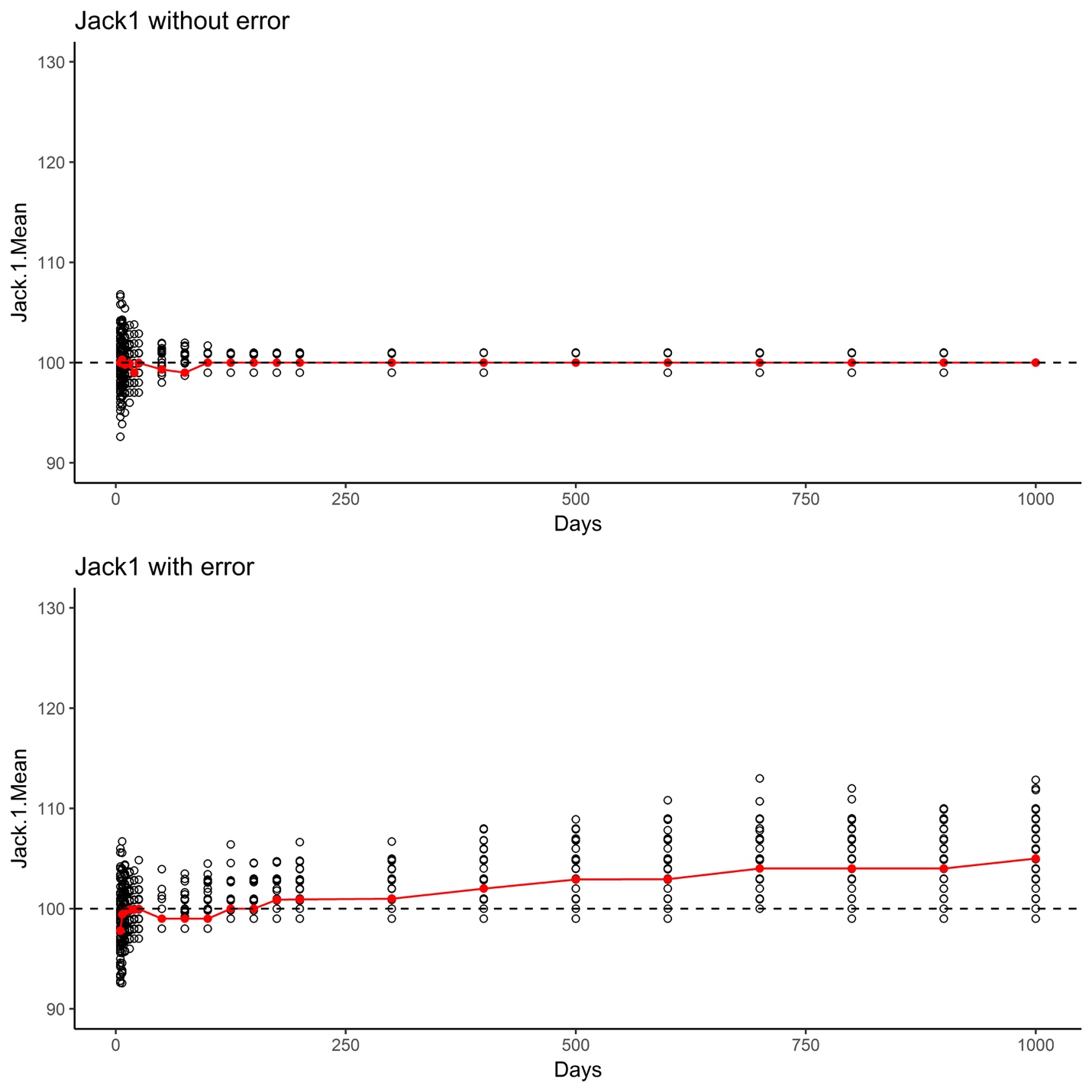

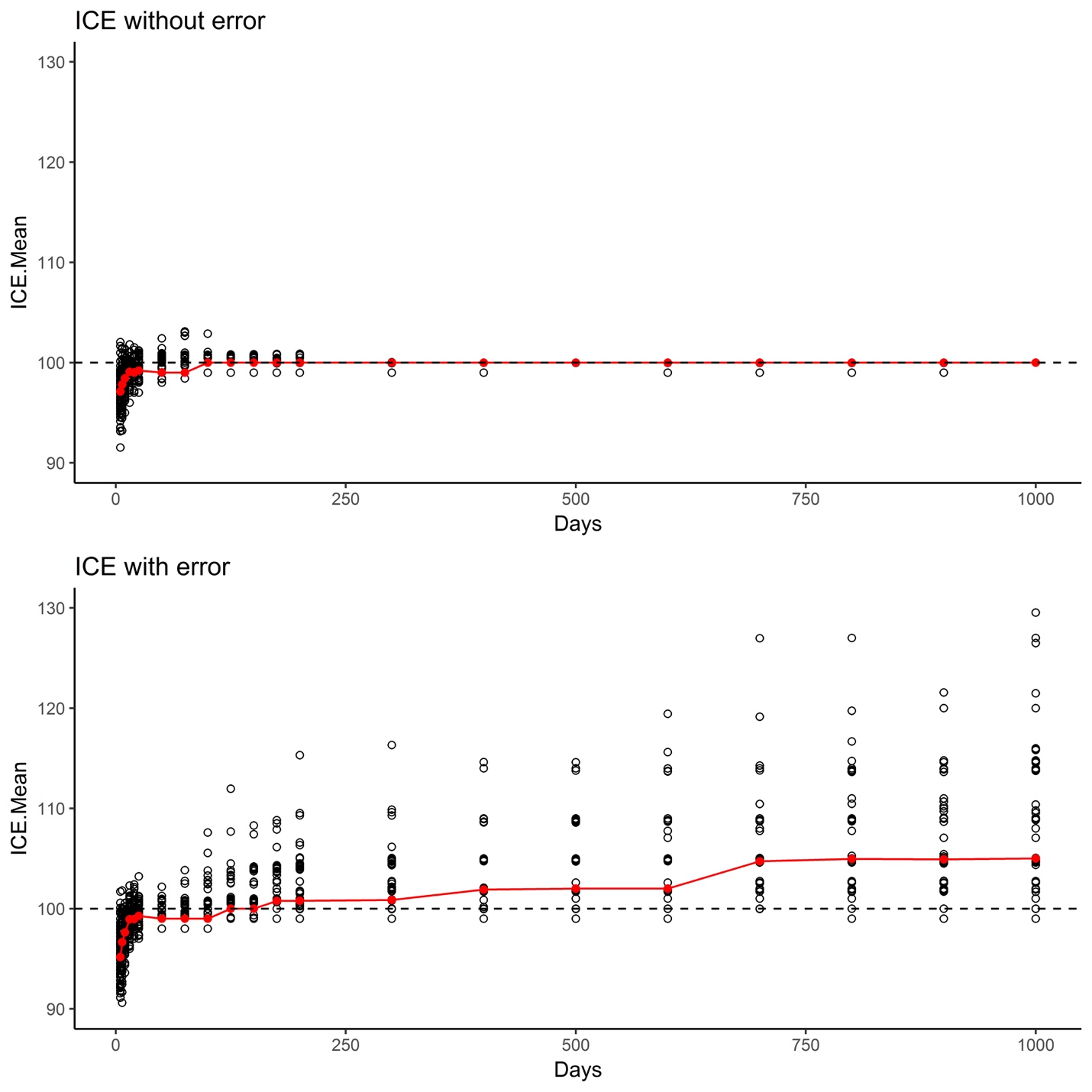


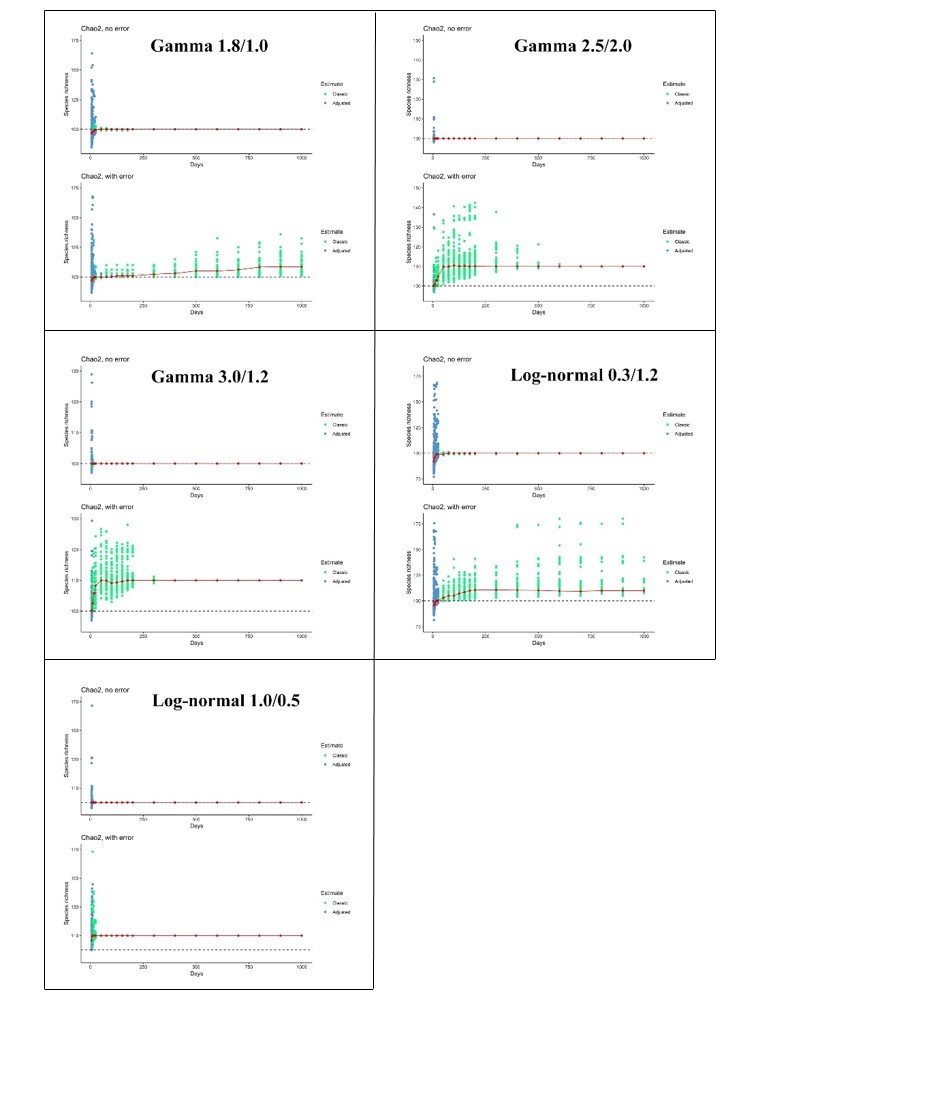
Summary of analyses of other abundance distributions, in estimations of species numbers in situations with and without error. The horizontal dashed line represents the “true” number of species—note that estimates in the “with error” in the log-normal distributions deviate markedly from the true numbers. Note that the blue dots refer to estimates from the Chiu-Chao estimator, whereas the green dots are situations in which the Chiu-Chao method defaults to the Chao2 estimator. The numbers presented in the label for each panel indicate parameters *µ* and $\sigma$ for log-normal distributions, and $\alpha$ and $\beta$ for gamma distributions.
